# Supplementary material for: Single-cell eQTL mapping of human endogenous retroviruses reveals cell type-specific genetic regulation in autoimmune diseases
Source: Nat Commun. 2025 Aug 14;16:7534. doi: 10.1038/s41467-025-62779-7 (PMC12354754; doi:10.1038/s41467-025-62779-7)
Supplement: Supplementary file 1 — Supplementary Information [file 41467_2025_62779_MOESM1_ESM.pdf]

Supplementary Figure 1

a

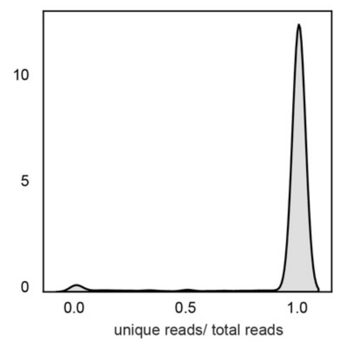

c

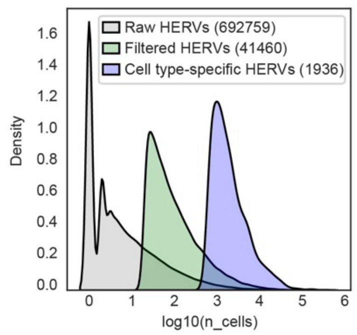

b

Methods	References	Mapping strategies	Throughput	Data Requirements	Resolution
Our Approach	DOI: 10.1016/j.ebiom.2022.104319	Retain only uniquely mapped reads	High (10000+ cell)	Short-read	Locus
soloTE	DOI: 10.1038/s42003-022-04020-5	Randomly assigns multi-mapping reads to HERV loci	High (10000+ cell)	Short-read	Locus
scTE	DOI: 10.1038/s41467-021-21808-x	Aggregates expression across all instances of a specific HERV family (e.g., LTR12C, MLT1A).	High (10000+ cell)	Short-read	Family
CELLO-Seq	DOI: 10.1038/s41587-021-01093-1	Integrates long-read sequencing with a bespoke computational framework.	Low (6-96 cell)	Long-read	Locus

Supplementary Figure1| Comprehensive Analysis of HERV Quantification: Distribution, Method Comparison, and Refinement Stages.

a, The kernel density plot illustrates the distribution of the percentage of unique reads mapped to HERVs. **b,** A comparative table summarizes the characteristics of our method and other approaches used in similar studies for HERV quantification. **c**, The kernel density plot illustrates the distribution of HERVs at three stages: Raw HERVs (initial unfiltered data), Filtered HERVs (filtering out HERVs expressed in fewer than 20 cells), and Cell type-specific HERVs (HERVs uniquely expressed in specific cell types, data derived from Fig. 3).

Supplementary Figure 2

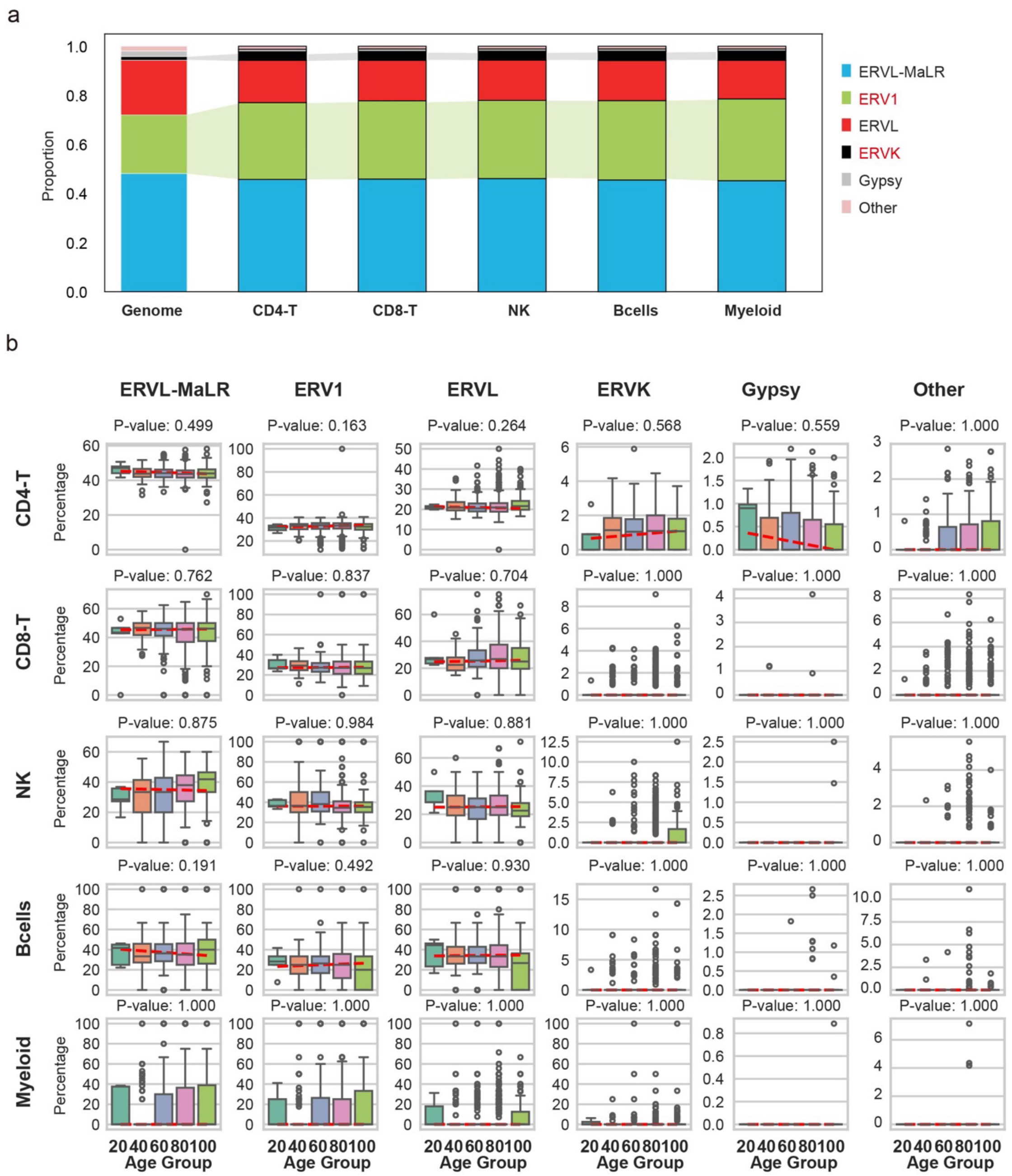

Supplementary Figure2| Distribution and Temporal Dynamics of HERV Families Across Cell Types.

a, Distribution of HERV families across five cell types. **b**, Temporal dynamics of HERV family proportions across each cell type. The p-value was derived by calculating the Pearson correlation coefficient.

Supplementary Figure 3

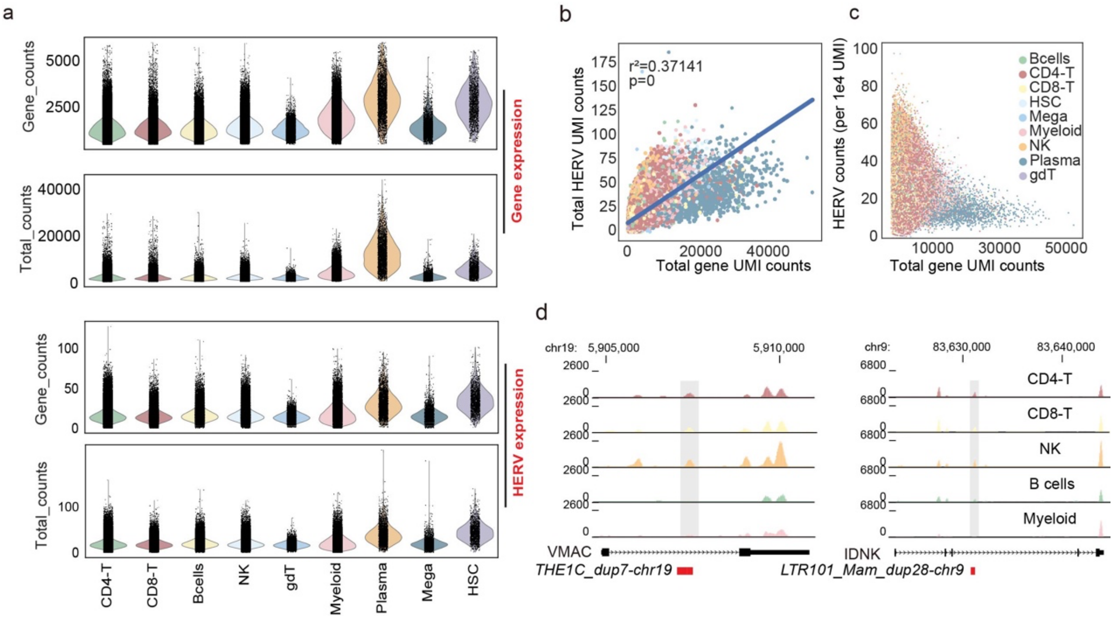

Supplementary Figure3| Normalization of HERVs.

a, Violin plot shows the distribution of gene counts, total gene counts, HERV counts, total HERV counts, and normalized HERV counts across different cell types. **b**, **c** The scatter plot illustrates the relationship between total gene UMI counts and total HERV UMI counts (b), and total normalized HERV counts (c), with each point colored by cell type. The fitted curve in b indicates a positive correlation between total gene UMI counts and total HERV UMI counts. And plot c indicates no correlation between total gene UMI counts and total normalized HERV counts. **d**, Distribution of RNA-seq peaks in the *IDNK* and *VMAC* loci across five cell types.

Supplementary Figure 4

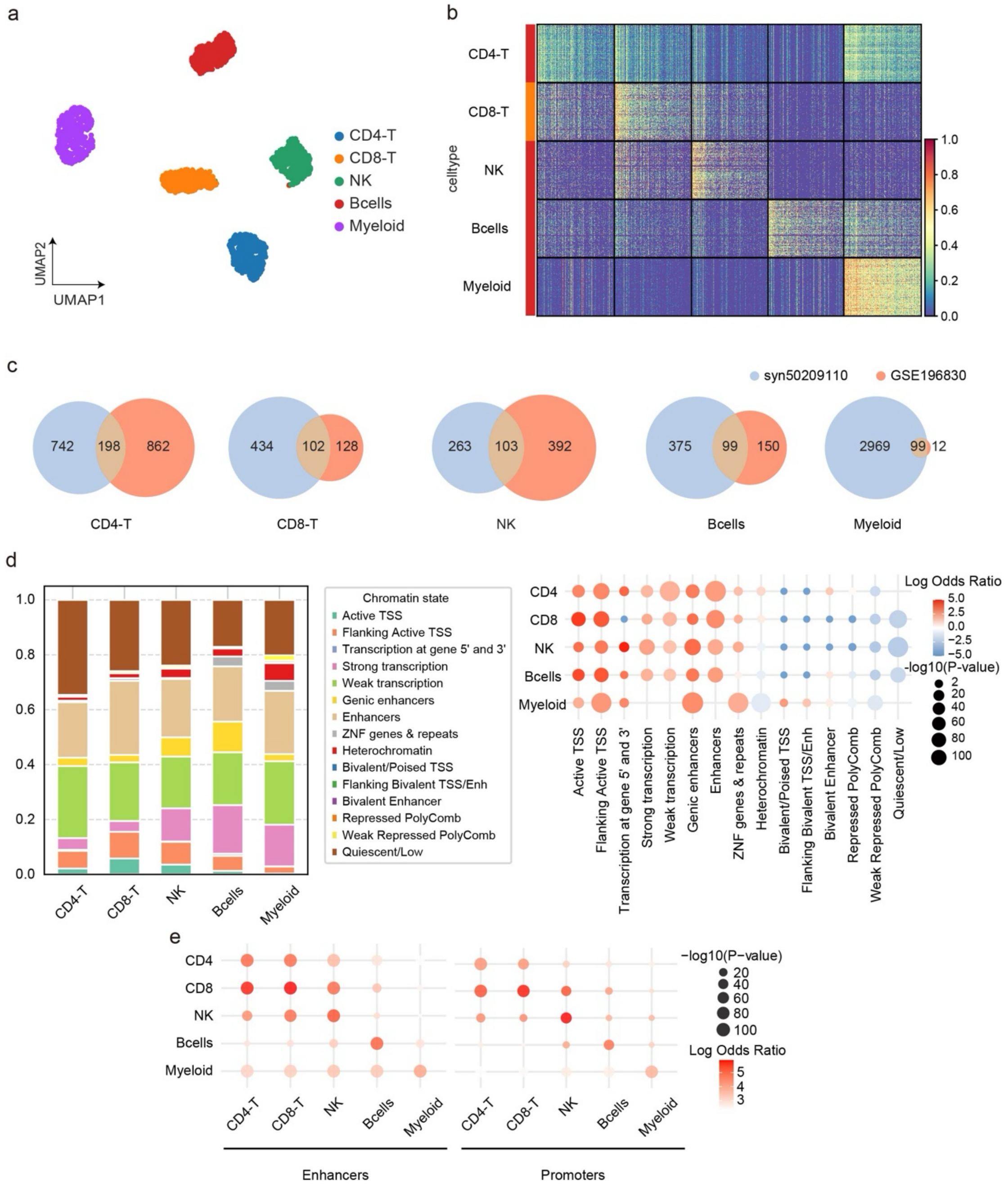

Supplementary Figure4| Validation of cell type-specific HERV expression in syn50209110.

a, UMAP projection based on the expression of highly variable HERVs in syn50209110[1]. Each spot represents a pseudobulk cell type sample of an individual. **b**, Heatmap of cell type-specific HERV expression. **c**, Overlap of cell type-specific HERV expression between the GSE196830 and syn50209110 datasets[1]. **d**, Chromatin state proportion and enrichment of cell type-specific HERV expression. **e**, Enrichment of

cell type-specific HERV expression in promoter and enhancer regions. P-value and Odds ratio was calculated using two-sided Fisher's exact tests comparing the observed overlap of HERVs with each promoter and enhancer regions against background expectations. Multiple testing correction was performed using the Benjamini-Hochberg false discovery rate (FDR) method (threshold = 0.05) implemented via the p.adjust function in R.

Supplementary Figure 5

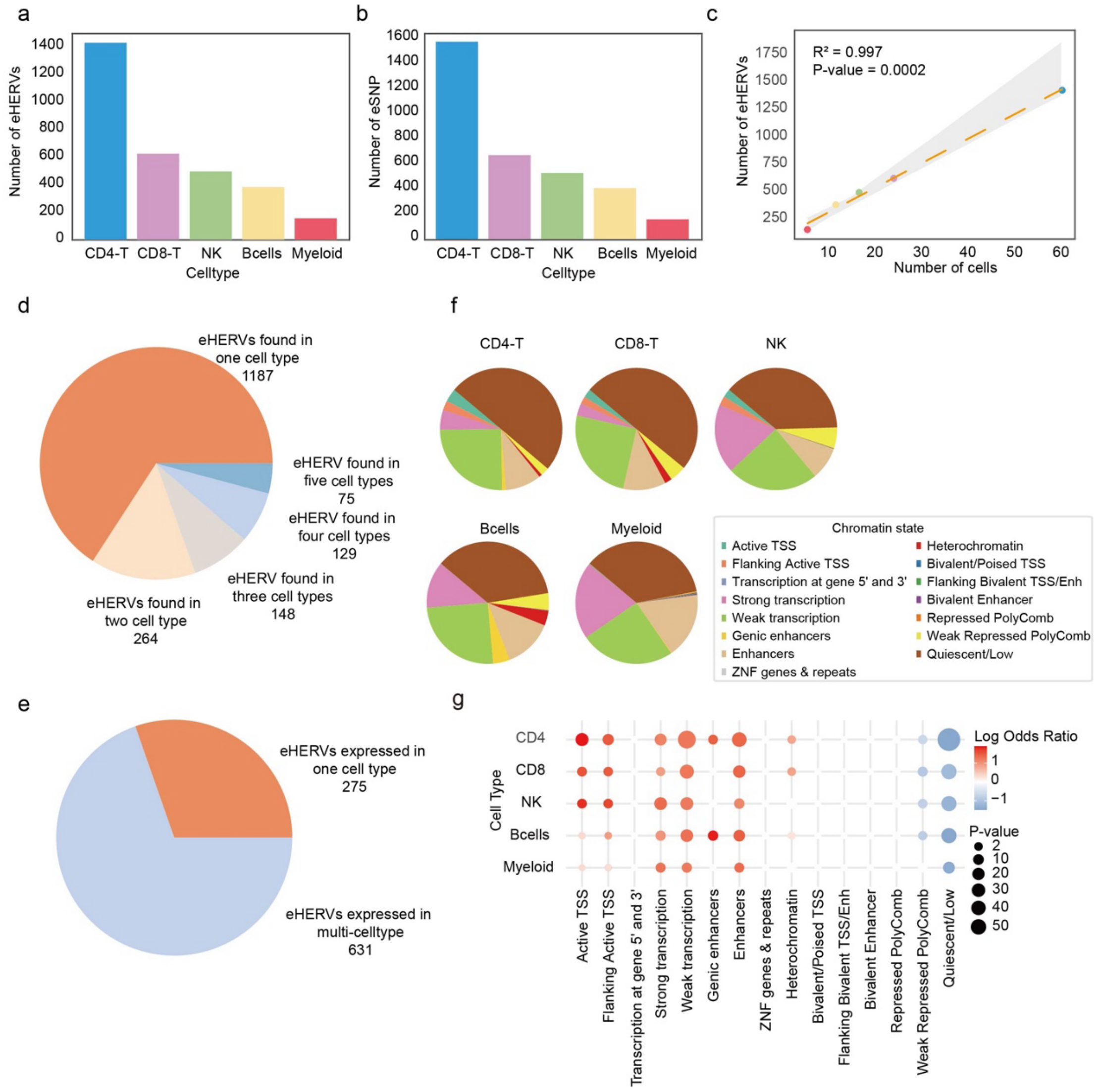

Supplementary Figure5| Characterization and chromatin context of eHERVs and eSNPs across cell types.

a, Bar plot showing the number of eHERVs in each cell type. **b**, Bar plot showing the number of eSNPs in each cell type. **c**, Scatter plot illustrating the Pearson's correlation between the number of cells and the number of eHERVs detected. The dashed line represents the least-squares linear regression fit, and the shaded area indicates the 95% confidence interval (CI) for the regression line. R denotes the Pearson correlation coefficient. **d**, Proportions of different types of eHERVs. **e**, Proportion of eHERVs found in one cell type that are expressed in either a single cell type or multiple cell types. **f**, Proportion of eSNPs across the 15 chromatin states corresponding to that cell type. chromatin states of immune cells were obtained from Roadmap Epigenomics[2]. **g**,

Enrichment of eHERVs within 15 chromatin state corresponding to that cell type. P-value and Odds ratio was calculated using two-sided Fisher's exact tests comparing the observed overlap of eSNPs with each chromatin state against background expectations. Multiple testing correction was performed using the Benjamini-Hochberg false discovery rate (FDR) method (threshold = 0.05) implemented via the p.adjust function in R.

Supplementary Figure 6

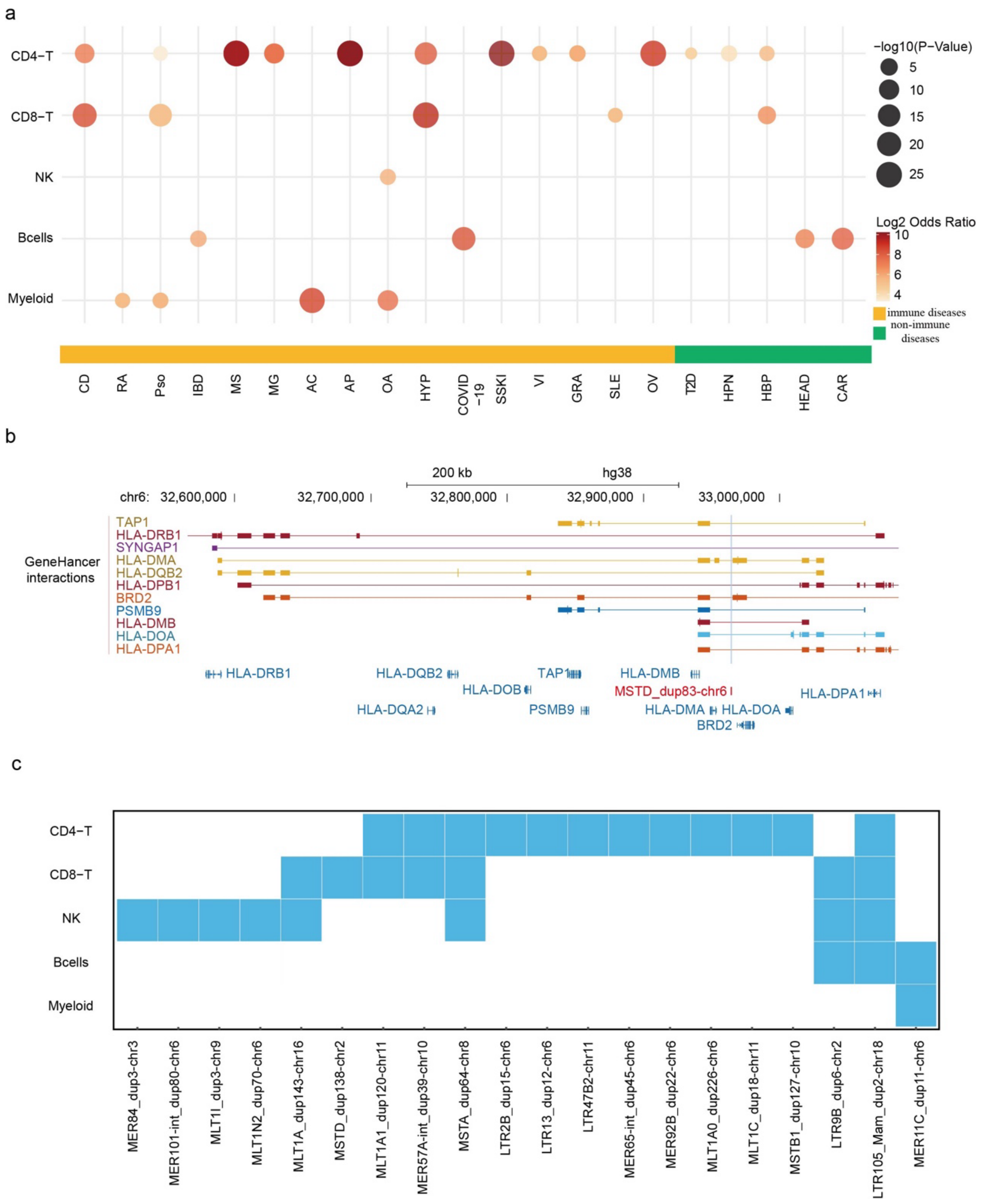

Supplementary Figure6| Association between HERVs and disease.

a, The enrichment of HERV-associated eSNPs identified from PBMCs in disease-associated SNPs from the GWAS catalog[3]. The bar at the bottom represents the type of disease. **b**, The top section of the figure displays interactions recorded in the GeneHancer database[4]. The x-axis denotes the physical position along a segment of chromosome 6 containing *MSTD_dup83-chr6* and the HLA genes that interact with *MSTD_dup83-chr6*. **c**, Heatmap presents a 0,1 matrix with HERVs represented along

the x-axis and cell types along the y-axis. Blue boxes indicate HERVs associated with CD (Crohn’s Disease), highlighting specific interactions between HERVs and cell types relevant to the disease.

Supplementary Figure 7

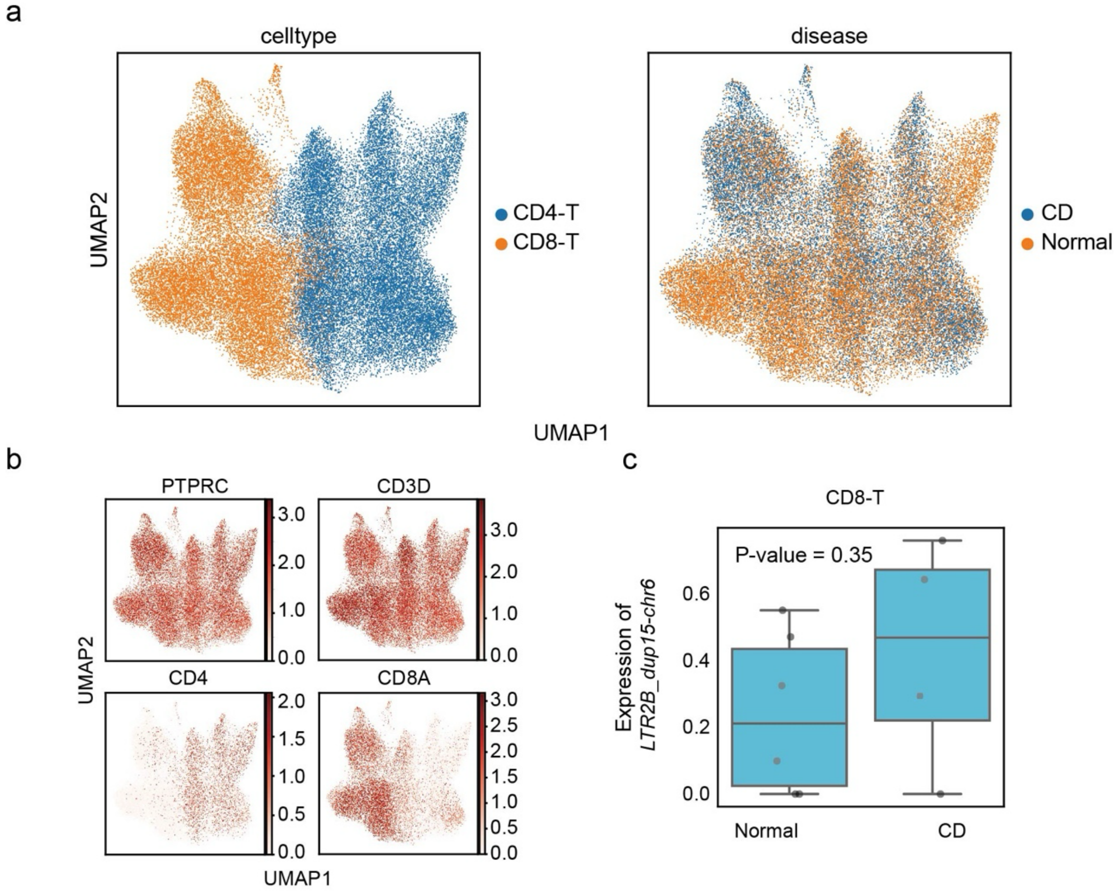

Supplementary Figure7| Analysis of disease-associated HERV expression in T cells from CD patients and healthy donors

a, Single-cell RNA-seq UMAP projection of T cells from CD patients and healthy donors[5]. Left: color coded to indicate the cell types. Right: color coded to indicate the disease state of the samples. **b**, UMAP projection of cell marker genes for CD4-T and CD8-T cells. **c**, Boxplots showing the expression of *LTR2B_dup15-chr6* in normal (left, n=6 biologically independent replicates) and patient (right, n=4 biologically independent replicates) CD8-T cells from CD single-cell data. Center lines represent medians, box limits indicate the 25th and 75th percentiles, whiskers extend to 1.5× interquartile range (IQR) from the box edges, and individual points show outliers. Statistical significance was assessed by two-tailed independent samples t-test.

Supplementary Figure 8

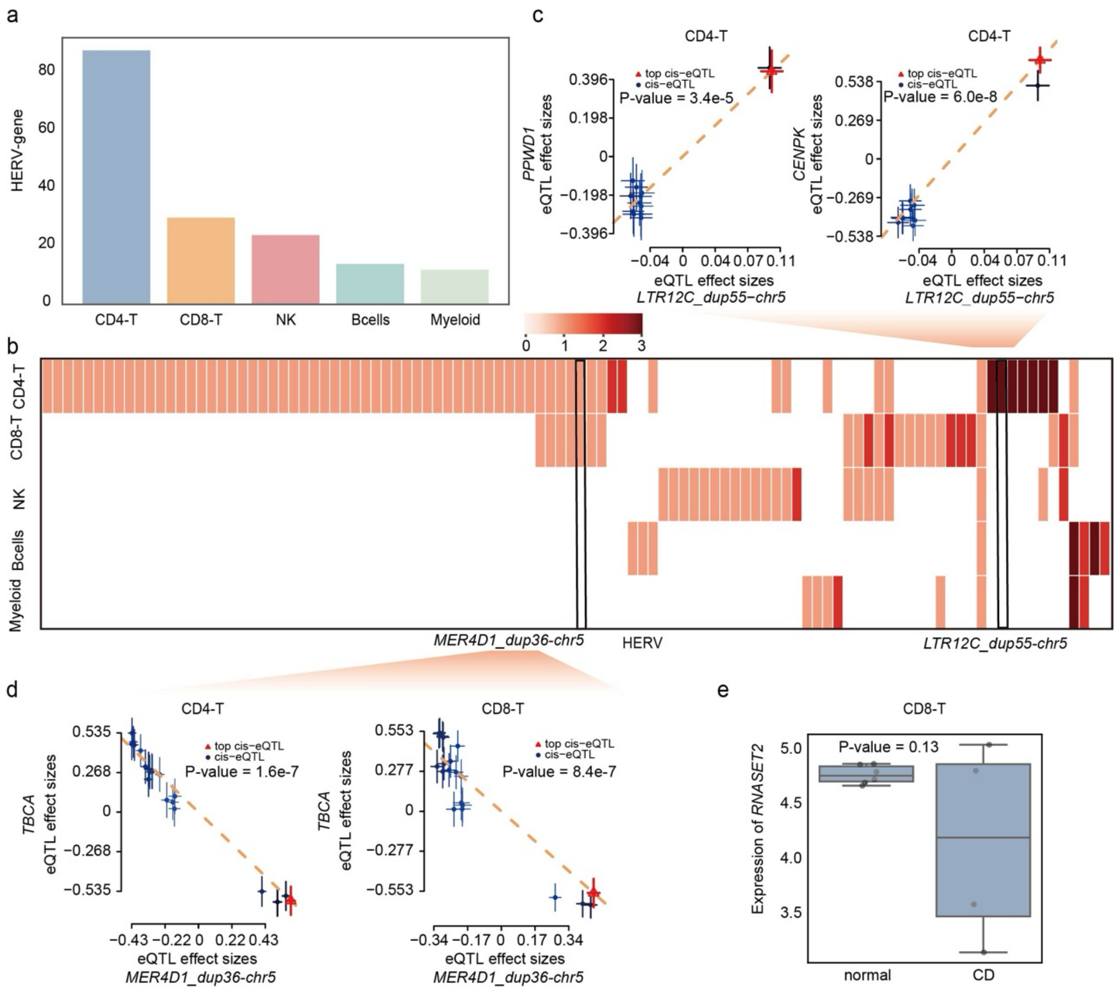

Supplementary Figure8| Pleiotropic associations of HERVs in gene expression.

a, Number of significant pleiotropic associations of HERVs with genes in each cell type. **b**, Number of significant pleiotropic associations of HERVs with genes across different cell types. The x-axis represents HERVs, and the y-axis denotes the cell types. Each entry in the matrix indicates the count of significant associations for a specific HERV within a particular cell type. **c**, Effect sizes of variants from the *LTR12C_dup55−chr5* eQTL plotted against those from the *PPWD1* and *CENPK* eQTL in CD4-T cells. P-value are derived from SMR analyses. Multiple testing correction was performed using the Benjamini-Hochberg false discovery rate (FDR) method (threshold = 0.05) implemented via the p.adjust function in R. The dashed lines represent the estimate of effect size at the top cis-eQTL. Error bars are the standard errors of SNP effects. **d**, Effect sizes of variants from the *MER4D1_dup36-chr5* eQTL plotted against those from the *TBCA* eQTL in CD4-T and CD8-T cells. P-value are derived from SMR analyses.

Multiple testing correction was performed using the Benjamini-Hochberg false discovery rate (FDR) method (threshold = 0.05) implemented via the p.adjust function in R. The dashed lines represent the estimate of effect size at the top cis-eQTL. Error bars are the standard errors of SNP effects. **e**, Boxplots showing the expression of *RNASET2* in normal (left, n=6 biologically independent replicates) and patient (right, n=4 biologically independent replicates) CD8-T cells from CD single-cell data. Center lines represent medians, box limits indicate the 25th and 75th percentiles, whiskers extend to 1.5× interquartile range (IQR) from the box edges, and individual points show outliers. Statistical significance was assessed by two-tailed independent samples t-test.

Reference

1. Terekhova, M. *et al.* Single-cell atlas of healthy human blood unveils age-related loss of NKG2C(+)GZMB(-)CD8(+) memory T cells and accumulation of type 2 memory T cells. *Immunity* **56(12)**, 2836-2854 (2023).
2. Kundaje, A. *et al.* Integrative analysis of 111 reference human epigenomes. *Nature* **518**, 317-330 (2015).
3. Sollis, E. *et al.* The NHGRI-EBI GWAS Catalog: knowledgebase and deposition resource. *Nucleic Acids Research* **51**, D977-D985 (2023).
4. Fishilevich, S. *et al.* GeneHancer: genome-wide integration of enhancers and target genes in GeneCards. *Database (Oxford)* **2017**(2017).
5. Jaeger, N. *et al.* Single-cell analyses of Crohn's disease tissues reveal intestinal intraepithelial T cells heterogeneity and altered subset distributions. *Nat Commun*, 1921 (2021).
