# Supplementary material for: Single-cell eQTL mapping of human endogenous retroviruses reveals cell type-specific genetic regulation in autoimmune diseases
Source: Nat Commun. 2025 Aug 14;16:7534. doi: 10.1038/s41467-025-62779-7 (PMC12354754; doi:10.1038/s41467-025-62779-7)
Supplement: Supplementary file 2 — Description of Additional Supplementary Files [file 41467_2025_62779_MOESM2_ESM.pdf]

Description of Additional Supplementary Files

Supplementary Data 1: Genome Coordinate Information of HERVs.

Supplementary Data 2: Genome Coordinate Information of HERVs Expressed in PBMCs.

Supplementary Data 3: Cell-Type Specific Expression of HERVs.

Supplementary Data 4: Summary Statistics for All eQTLs Identified by TensorQTL Conditional eQTL Analysis.

Supplementary Data 5: GWAS data for 81 immune-related diseases

Supplementary Data 6: Summary of SMR Analysis Results Linking HERVs to 81 Immune-Associated Traits.

Supplementary Data 7: Summary of SMR Analysis Results Linking HERVs to Genes in PBMCs

Supplementary Data 8: Summary of SMR Analysis Results Linking Genes to 81 Immune-Associated Traits.
