# Supplementary material for: Single-cell eQTL mapping of human endogenous retroviruses reveals cell type-specific genetic regulation in autoimmune diseases
Source: Nat Commun. 2025 Aug 14;16:7534. doi: 10.1038/s41467-025-62779-7 (PMC12354754; doi:10.1038/s41467-025-62779-7)
Supplement: Supplementary file 4 — Reporting Summary [file 41467_2025_62779_MOESM4_ESM.pdf]

nature portfolio

Corresponding author(s): Young Li
Last updated by author(s): Jul 19, 2025

# Reporting Summary

Nature Portfolio wishes to improve the reproducibility of the work that we publish. This form provides structure for consistency and transparency in reporting. For further information on Nature Portfolio policies, see our Editorial Policies and the Editorial Policy Checklist.

## Statistics

For all statistical analyses, confirm that the following items are present in the figure legend, table legend, main text, or Methods section.

| n/a | Confirmed | |
|---|---|---|
| [ ] | [x] | The exact sample size ($n$) for each experimental group/condition, given as a discrete number and unit of measurement |
| [ ] | [x] | A statement on whether measurements were taken from distinct samples or whether the same sample was measured repeatedly |
| [ ] | [x] | The statistical test(s) used AND whether they are one- or two-sided<br>*Only common tests should be described solely by name; describe more complex techniques in the Methods section.* |
| [ ] | [x] | A description of all covariates tested |
| [ ] | [x] | A description of any assumptions or corrections, such as tests of normality and adjustment for multiple comparisons |
| [ ] | [x] | A full description of the statistical parameters including central tendency (e.g. means) or other basic estimates (e.g. regression coefficient) AND variation (e.g. standard deviation) or associated estimates of uncertainty (e.g. confidence intervals) |
| [ ] | [x] | For null hypothesis testing, the test statistic (e.g. $F$, $t$, $r$) with confidence intervals, effect sizes, degrees of freedom and $P$ value noted<br>*Give P values as exact values whenever suitable.* |
| [x] | [ ] | For Bayesian analysis, information on the choice of priors and Markov chain Monte Carlo settings |
| [x] | [ ] | For hierarchical and complex designs, identification of the appropriate level for tests and full reporting of outcomes |
| [ ] | [x] | Estimates of effect sizes (e.g. Cohen's $d$, Pearson's $r$), indicating how they were calculated |

*Our web collection on statistics for biologists contains articles on many of the points above.*

## Software and code

Policy information about availability of computer code

| | |
|---|---|
| Data collection | cellranger (v7.1.0) |
| Data analysis | python (version 3)Scanpy (version 1.9.8) SciPy (version 1.12.0) deepTools49 (version 1.12.0) epitools (version 0.5-10.1) TensorQTL (version 1.0.8) SMR (version 1.3.1) R (version4) coloc (version v.5.2.3) fenrichcpp (version v1.1)The custom code for all analyses presented in this study is available on GitHub (https://github.com/YoungLi88/HERV_eQTL). |

For manuscripts utilizing custom algorithms or software that are central to the research but not yet described in published literature, software must be made available to editors and reviewers. We strongly encourage code deposition in a community repository (e.g. GitHub). See the Nature Portfolio guidelines for submitting code & software for further information.

## Data

Policy information about availability of data

All manuscripts must include a data availability statement. This statement should provide the following information, where applicable:

- Accession codes, unique identifiers, or web links for publicly available datasets
- A description of any restrictions on data availability
- For clinical datasets or third party data, please ensure that the statement adheres to our policy

Single-cell gene expression and genotype data from the OneK1K cohort are available through the Gene Expression Omnibus (https://www.ncbi.nlm.nih.gov/geo/query/acc.cgi?acc=GSE196830). The validation PBMC single cell RNA-seq dataset can be obtained from Synapse (https://www.synapse.org/Synapse:syn50209110). The 15 chromatin state predictions for each immune cell types can be downloaded from the Roadmap Epigenomics Project (https://egg2.wustl.edu/roadmap/

web_portal/chr_state_learning.html#core_15state). Histone ChIP-seq (H3K27ac, H3K4me3) and Dnase-seq for five immune cell types are available through the ENCODE project (https://www.encodeproject.org/). Summary statistics for 81 diseases (Supplementary Table 65) can be downloaded from the GWAS Catalog (https://www.ebi.ac.uk/gwas/).Single-cell gene expression data for CD are available through the Gene Expression Omnibus (https://www.ncbi.nlm.nih.gov/geo/query/acc.cgi?acc= GSE157477). Source data are provided with this paper.

## Research involving human participants, their data, or biological material

Policy information about studies with human participants or human data. See also policy information about sex, gender (identity/presentation), and sexual orientation and race, ethnicity and racism.

| | |
|---|---|
| Reporting on sex and gender | Most findings of this study will apply to both male and female because cis-eQTL mapping was performed using participants of both sex while adjusting for sex as a covariate. There are 416 males and 565 females.<br>Disaggregated sex of participants can be found at https://www.ncbi.nlm.nih.gov/geo/query/acc.cgi?acc=GSE196830 |
| Reporting on race, ethnicity, or other socially relevant groupings | The samples were all European, as described in the article from which the data were derived (OneK1K study). |
| Population characteristics | Population characteristics are described in detail in the original publication (https://www.science.org/doi/10.1126/science.abf3041). All samples were derived from healthy individuals. |
| Recruitment | This study utilized publicly available datasets (OneK1K study) and did not involve direct recruitment of participants. |
| Ethics oversight | This study utilized publicly available datasets (OneK1K study) and therefore has no ethical implications. |

Note that full information on the approval of the study protocol must also be provided in the manuscript.

# Field-specific reporting

Please select the one below that is the best fit for your research. If you are not sure, read the appropriate sections before making your selection.

[x] Life sciences  [ ] Behavioural & social sciences  [ ] Ecological, evolutionary & environmental sciences

For a reference copy of the document with all sections, see nature.com/documents/nr-reporting-summary-flat.pdf

# Life sciences study design

All studies must disclose on these points even when the disclosure is negative.

| | |
|---|---|
| Sample size | No sample size calculation was performed. Our sample size (n = 981) will be sufficient because typical eQTL study requires at least 100 samples. |
| Data exclusions | The data used for our analysis were downloaded from public sources, and no data were excluded from the analyses. |
| Replication | For statistically significant findings, we looked for evidence in multiple dataset used in this study.<br>To validate the enrichment of cell type-specific HERVs in active chromatin regions, we repeated these analyses using another single-cell RNA-seq dataset of PBMCs (syn50209110). Consistently, we observed cell type-specific HERV profiles that were significantly shared between the two datasets (Supplementary Fig. 3a-3c). The cell type-specific HERVs were also enriched in the active chromatin regions of corresponding cell types (Supplementary Fig. 3d-3e).<br>To verify SMR association, we conducted genetic colocalization analysis, which tests whether two potentially related phenotypes share common genetic causal variants in a given genomic region.<br>We further compared the expression of LTR2B_dup15-chr6 in single-cell RNA-seq data from CD patients and healthy donors (GSE157477), which showed that LTR2B_dup15-chr6 was significantly expressed in CD4-T cells of CD patients, but not in other cell types (Fig. 5g, Supplementary Fig. 7a-7c), indicating that LTR2B_dup15-chr6 might be a CD4-T cell type-specific genetic causal effect of CD.<br>all attempts at replication were successful. |
| Randomization | Randomization is not relevant because this is an observational study. |
| Blinding | Blinding was not applicable to this study as all data were obtained from publicly available datasets. The investigators did not participate in the original data collection or group allocation processes, and the analysis was conducted solely based on the pre-existing data. Therefore, blinding was neither possible nor relevant to the study design. |

# Reporting for specific materials, systems and methods

We require information from authors about some types of materials, experimental systems and methods used in many studies. Here, indicate whether each material, system or method listed is relevant to your study. If you are not sure if a list item applies to your research, read the appropriate section before selecting a response.

## Materials & experimental systems

| n/a | Involved in the study |
|---|---|
| ☒ | ☐ Antibodies |
| ☒ | ☐ Eukaryotic cell lines |
| ☒ | ☐ Palaeontology and archaeology |
| ☒ | ☐ Animals and other organisms |
| ☒ | ☐ Clinical data |
| ☒ | ☐ Dual use research of concern |
| ☒ | ☐ Plants |

## Methods

| n/a | Involved in the study |
|---|---|
| ☒ | ☐ ChIP-seq |
| ☒ | ☐ Flow cytometry |
| ☒ | ☐ MRI-based neuroimaging |

# Plants

| | |
|---|---|
| Seed stocks | *Report on the source of all seed stocks or other plant material used. If applicable, state the seed stock centre and catalogue number. If plant specimens were collected from the field, describe the collection location, date and sampling procedures.* |
| Novel plant genotypes | *Describe the methods by which all novel plant genotypes were produced. This includes those generated by transgenic approaches, gene editing, chemical/radiation-based mutagenesis and hybridization. For transgenic lines, describe the transformation method, the number of independent lines analyzed and the generation upon which experiments were performed. For gene-edited lines, describe the editor used, the endogenous sequence targeted for editing, the targeting guide RNA sequence (if applicable) and how the editor was applied.* |
| Authentication | *Describe any authentication procedures for each seed stock used or novel genotype generated. Describe any experiments used to assess the effect of a mutation and, where applicable, how potential secondary effects (e.g. second site T-DNA insertions, mosiacism, off-target gene editing) were examined.* |
